# GWAS of five gynecologic diseases and cross-trait analysis in Japanese

**DOI:** 10.1038/s41431-019-0495-1

**Published:** 2019-09-05

**Authors:** Tatsuo Masuda, Siew-Kee Low, Masato Akiyama, Makoto Hirata, Yutaka Ueda, Koichi Matsuda, Tadashi Kimura, Yoshinori Murakami, Michiaki Kubo, Yoichiro Kamatani, Yukinori Okada

**Affiliations:** 1 Department of Statistical Genetics, Osaka University Graduate School of Medicine, Suita, 565-0871 Japan; 2 Department of Obstetrics and Gynecology, Osaka University Graduate School of Medicine, Osaka, 565-0871 Japan; 3 Laboratory for Statistical Analysis, RIKEN Center for Integrative Medical Sciences, Yokohama, 230-0045 Japan; 4 Cancer Precision Medicine Center, Japanese Foundation for Cancer Research, Tokyo, 135-8550 Japan; 5 Department of Ophthalmology, Graduate School of Medical Sciences, Kyushu University, Fukuoka, Fukuoka 812-8582 Japan; 6 Laboratory of Genome Technology, Institute of Medical Science, the University of Tokyo, Tokyo, 108-8639 Japan; 7 Department of Computational Biology and Medical Sciences, Graduate school of Frontier Sciences, The University of Tokyo, Tokyo, 108-8639 Japan; 8 Division of Molecular Pathology, the Institute of Medical Sciences, the University of Tokyo, Tokyo, 108-8639 Japan; 9 RIKEN Center for Integrative Medical Sciences, Yokohama, 230-0045 Japan; 10 Kyoto-McGill International Collaborative School in Genomic Medicine, Graduate School of Medicine, Kyoto University, Kyoto, 606-8507 Japan; 11 Laboratory of Statistical Immunology, Immunology Frontier Research Center (WPI-IFReC), Osaka University, Suita, 565-0871 Japan

**Keywords:** Genome-wide association studies, Genetics research

## Abstract

We performed genome-wide association studies of five gynecologic diseases using data of 46,837 subjects (5236 uterine fibroid, 645 endometriosis, 647 ovarian cancer (OC), 909 uterine endometrial cancer (UEC), and 538 uterine cervical cancer (UCC) cases allowing overlaps, and 39,556 shared female controls) from Biobank Japan Project. We used the population-specific imputation reference panel (*n* = 3541), yielding 7,645,193 imputed variants. Analyses performed under logistic model, linear mixed model, and model incorporating correlations identified nine significant associations with three gynecologic diseases including four novel findings (rs79219469:C > T, *LINC02183*, *P* = 3.3 × 10^−8^ and rs567534295:C > T, *BRCA1*, *P* = 3.1 × 10^−8^ with OC, rs150806792:C > T, *INS-IGF2*, *P* = 4.9 × 10^−8^ and rs140991990:A > G, *SOX9*, *P* = 3.3 × 10^−8^ with UCC). Random-effect meta-analysis of the five GWASs correcting for the overlapping subjects suggested one novel shared risk locus (rs937380553:A > G, *LOC730100*, *P* = 2.0 × 10^−8^). Reverse regression analysis identified three additional novel associations (rs73494486:C > T, *GABBR2*, *P* = 4.8 × 10^−8^, rs145152209:A > G, *SH3GL3*/*BNC1*, *P* = 3.3 × 10^−8^, and rs147427629:G > A, *LOC107985484*, *P* = 3.8 × 10^−8^). Estimated heritability ranged from 0.026 for OC to 0.220 for endometriosis. Genetic correlations were relatively strong between OC and UEC, endometriosis and OC, and uterine fibroid and OC (*r*_*g*_ > 0.79) compared with relatively weak correlations between UCC and the other four (*r*_*g*_ = −0.08 ~ 0.25). We successfully identified genetic associations with gynecologic diseases in the Japanese population. Shared genetic effects among multiple related diseases may help understanding the pathophysiology.

## Introduction

Uterine fibroma (UF), endometriosis, ovarian cancer (OC), uterine endometrial cancer (UEC), and uterine cervical cancer (UCC) are all common proliferative diseases arising from gynecologic organs. They are heterogeneous diseases with diverse range of proliferative and infiltrative properties. Clinical and epidemiological studies suggest that these diseases are mutually associated or often occur as comorbidity [1]. Studies of shared background risk, namely genetics, would offer understanding of the causes of these diseases, along with identifying targets to be treated.

For the past several years, genetic studies of gynecologic diseases have revealed only a limited number of significant associations [2–10]. Of note, the common risk genes well-known from pedigree studies, such as *BRCA1* and *BRCA2* [11], have not been reported as either ovarian or breast cancer-susceptibility genes in the context of genome-wide association study (GWAS) [7, 12]. This is largely because risk variants found in pedigree studies are usually rare among general population, which is unlikely detected in GWAS. So we focused on variants common among general population, including low frequency ones, to assess if these risk genes impose risks to general population, not just to specific families. The majority of the associations found in GWAS have small effect and polygenic nature. Detection of genetic associations in such cases depends on large sample size and ingenious analytical strategies. Genetic studies in the field of gynecology, including those reported from Biobank Japan Project (BBJ) [2, 4, 13], have been performed under the common logistic regression model. In this paper, to facilitate the detection of association signals and generalize the results, we conducted association studies under the liner mixed model (BOLT-LMM), with the largest GWAS data of Japanese population to date from BBJ. Advantages in adopting mixed models include that (i) they could account for both population stratification and cryptic relatedness, (ii) they could avoid confounding factors and provide robust association results, and (iii) they achieve increased statistical power for identifying genetic associations [14]. Large computational burden, a major limitation in mixed model methods especially when sample size is large, is overcome in BOLT-LMM [14]. In order to further enhance detection of novel loci, we also tried multiple association analytic approaches, where correlations between GWAS estimates among multiple related diseases are incorporated using MTAG [15]. Although MTAG utilizes summary level data and comparison between analyses based on row genotype data and summary level data might not be straightforward, the MTAG results were comparable to those from analyses under the common logistic model (mach2dat) and linear mixed model (BOLT-LMM) in a disease-specific manner. Since MTAG utilizes bivariate linkage disequilibrium (LD) score regression, where linear regression with liability threshold model is assumed and regression *z*-scores are assumed to follow standard normal distribution, which is different from the linear mixed model and non-normal distribution of regression z-scores assumed in BOLT-LMM, we utilized the results of mach2dat for applying MTAG. Also, we applied the reverse regression model using SCOPA [16], which utilizes raw genotype data, and produces estimates based on the best combination of phenotypes fitted to the model to obtain the maximized log-likelihood. We considered that SCOPA results could be comparable to those of joint analysis of all cases versus shared controls and random effect GWAS meta-analysis of different diseases in a multiple-disease-combined manner.

Clinically and epidemiologically, some of the gynecologic disorders harbor shared risk factors, such as age at menarche or menopause, and body mass index (BMI) [17–19]. These risk factors are at least partially influenced genetically and might be under the shared or pleiotropic effects of the genome [20]. To our knowledge, limited studies have investigated the shared genetic effects on gynecologic diseases, and they specifically look into relationship between two of the gynecologic diseases [21, 22], relationship among histologic subtypes [23], or include gynecologic diseases as a part of multi-disease/trait study [24]. Only one of these studies analyzed the genetic correlation between specific pair of diseases under the linear mixed model [21]. To increase our understanding of shared genetic determinants influencing gynecologic diseases, here we report genetic correlations among the five gynecologic diseases in Japanese population using the linear mixed model approach.

## Subjects and methods

### Subjects

In total, 7315 cases with one of the clinically diagnosed five gynecologic diseases, including UC, endometriosis, OC, UEC, and UCC including cervical intraepithelial neoplasias, and 39,829 shared female controls without gynecologic diseases were enrolled from BBJ [13]. All the subjects provided written informed consent as approved by the ethical committee of RIKEN Yokohama Institute and the Institute of Medical Science, the University of Tokyo. This study was approved by the ethical committee of Osaka University Graduate School of Medicine. Females affected with non-gynecologic malignancies and/or diseases thought to be strongly associated with the major histocompatibility complex (MHC) region were excluded from the control group. Related subjects were excluded in advance to avoid possible confounding. Carrier status of known risk genes such as *BRCA1* and *BRCA2*, histopathological subtypes, and disease severity such as tumor sizes and clinical stages were not considered. Principal component analysis (PCA) was performed using EIGENSOFT (v6.1.4) Data manipulation was performed using PLINK software (v1.90b3.3).

### Genotype imputation

Genotypes of the samples were obtained using either of the following genotyping arrays: (i) the Illumina HumanOmniExpressExome BeadChip or (ii) a combination of the Illumina HumanOmniExpress and HumanExome BeadChips. Genotype data is deposited on the Japanese Genotype-phenotype Archive affiliated to the DNA Data Bank of Japan, via National Bioscience Database Center, Japan. The data are accessible on hum0014 at https://ddbj.nig.ac.jp/jga/viewer/view/study/JGAS00000000114. For quality control (QC) of genotypes, we excluded variants meeting any of the following criteria: (i) call rate < 99%, (ii) *P*-value for departure from Hardy–Weinberg equilibrium (HWE) < 1.0 × 10^−6^, and (iii) number of heterozygotes less than five.

After we proceeded through these QC steps, we used Eagle (v2.3) for haplotype phasing without an external reference panel. We conducted whole-genome imputation using Minimac3 (v1.0.11) and the population-specific reference panel including multi-ethnic 2504 samples in 1000 Genomes Project (1KGP) phase 3v5a and deep whole genome sequencing of 1037 Japanese [25, 26]. Variants with minor allele frequency (MAF) ≥ 1% in both case and control subjects, and imputation info *r*^2^ ≥ 0.7 were selected for the following analyses.

### Association analyses

Associations of the variants with each disease were separately evaluated (i) under the logistic regression model assuming additive effects of the allele dosages using mach2dat (v1.0.24), (ii) under the linear mixed model using BOLT(-LMM) (v2.2) [14], and (iii) by incorporating correlations between GWAS summary statistics using MTAG (v1.0.7) [15] with mach2dat results. Age, squared age, BMI, and the top 20 principal components (PCs) were included as covariates. PCs were calculated from 175,574 genotyped variants using EIGENSOFT (v6.1.4) under the same QC and pruning after excluding PCA outliers as described below in Heritability and genetic correlation section.

Joint analysis of all cases versus shared controls was executed under the logistic regression model using mach2dat, and was compared with random-effect meta-analysis of mach2dat results of each disease using RE2C (v1.04) [27] to correct for the overlapping samples and increase the power for detection. The same set of analyses was also conducted under the linear mixed model using BOLT-LMM and compared with the corresponding mach2dat results. We also applied the reverse regression model to the imputed genotypes and covariates-adjusted phenotypes using SCOPA (v1.0.14) [16]. The SCOPA results were compared with those from above analyses. Since RE2C* *P*-value, which is conditioned for the overlapping samples but is not conditioned for the fixed effect, was used for plotting, genome-wide significance threshold was defined as *P* = 5.0 × 10^−8^ for all analyses [28]. Metasoft (v2.0.1) was applied to calculate meta-analysis heterogeneity index *I*^2^.

### Candidate gene and functional annotation

For gynecologic risk loci outside of the MHC region, candidate genes were prioritized by FUMA [29]. We used the default settings except for the followings; we set the LD r^2^ threshold to 0.5 to define LD structure of lead SNPs, reference panel population to 1KGP phase 3 EAS, and minimum allele frequency to 0.004. When there are no “mapped genes” then we listed the nearest coding or noncoding genes. Since genetic architecture within the MHC region is complex [30], it is displayed as “the MHC region” instead of the gene names. Functional annotations of the identified variants were also obtained by applying GARFIELD [31] and searching through HaploReg v4.1 (see URLs).

### Heritability and genetic correlation

Heritability and genetic correlation were estimated using GCTA-GREML and phenotype-correlation-genotype-correlation (PCGC-s) (v1.0.0) [32], respectively, with the same genotyped variant matrix, which passed the QC criteria as follows; we excluded samples with call rate < 99%, variants with call rate < 99%, variants with MAF < 5%, variants with HWE *P*-value < 1.0 × 10^−6^, variants in the MHC with flanking region spanning from 24 to 34 Mb of chromosome 6, then pruned variants at LD *r*^2^ cutoff of 0.50. Disease prevalence was defined as described elsewhere [1, 33, 34]. Age, squared age, BMI, and 20 PCs were incorporated as covariates. BOLT-REML failed to converge in analyzing heritability, and GCTA-GREML failed to converge in analyzing genetic correlation, therefore we adopted PCGC-s that would definitely yield the results through Haseman–Elston regression model. PCGC-s does not produce standard errors or *p*-values for the estimates.

## Results

### Subjects

Numbers of the subjects eligible for each GWAS were as follows; 5236 for UF, 645 for endometriosis, 647 for OC, 909 for UEC, and 538 for UCC cases where those who have multiple diseases were allowed to enroll in each corresponding GWAS, and 39,556 shared female controls. Characteristics of the samples included in the GWASs of the five gynecologic diseases are shown in Table 1.

**Table 1:** Characteristics of the genome-wide association studies of five gynecologic diseases

Diseases	No. cases	No. controls^a^	Disease prevalence^b^	*h*^*2*^_*g*_^c^	*h*^*2*^_*SNP*_^d^ (SE)
Uterine fibroid	5 236	39 556	1.89 × 10^−1^	0.26	1.70 × 10^−1^ (2.57 × 10^−2^)
Endometriosis	645	39 556	6.94 × 10^−2^	0.47–0.51	2.20 × 10^−1^ (1.30 × 10^−1^)
Ovarian cancer	647	39 556	1.22 × 10^−2^	0.40	2.60 × 10^−2^ (7.95 × 10^−2^)
Uterine endometrial cancer	909	39 556	1.64 × 10^−2^	0.52	1.26 × 10^−1^ (6.19 × 10^−2^)
Uterine cervical cancer	538	39 556	1.28 × 10^−2^	0.11–0.34	1.17 × 10^−1^ (9.75 × 10^−2^)

The numbers are rounded to three significant digits for disease prevalence and *h*^*2*^*SNP*

^a^Shared female controls among the five GWAS

^b^Disease prevalence among Japanese population

^c^Heritability previously estimated from twin, family or population-based case-control studies

^d^Heritability estimated from the GWAS data adjusted for disease prevalence

### Genotype imputation

Whole-genome imputation yielded 61,608,817 variants in total. Of these variants, 7,645,193 passed the quality control as described above (MAF ≧ 1% in both cases and controls, and imputation info *r*^2^ ≧ 0.70).

### Association analyses for single disease

**Fig. 1:**
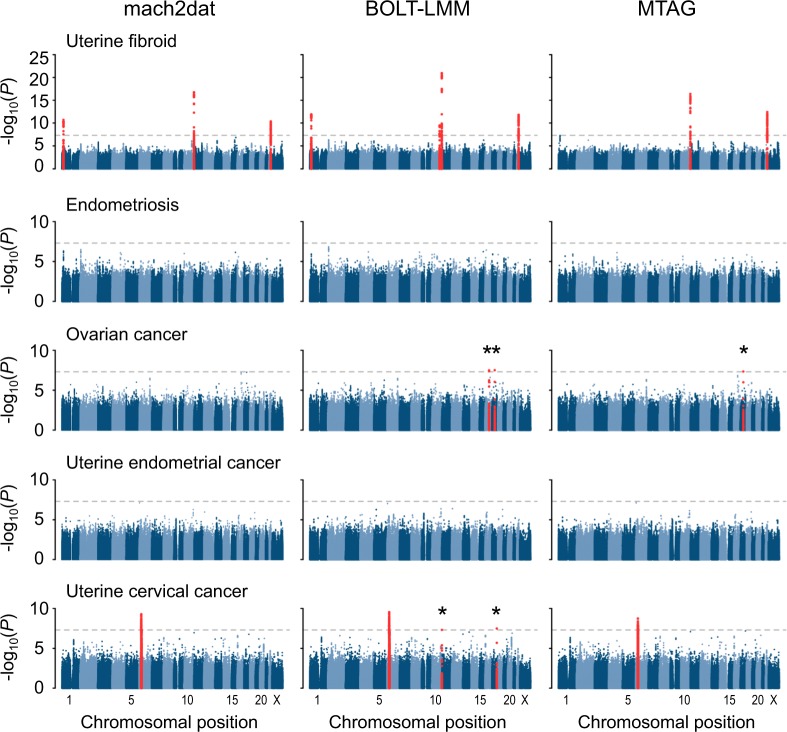
Manhattan plots of the five GWASs of gynecologic diseases. Manhattan plots of the GWAS of the five gynecologic diseases among Japanese. The *y*-axis indicates -log_10_(*P*) of association of each variant calculated by three methods, including the logistic regression model using mach2dat, the linear mixed model using BOLT-LMM, and incorporating correlations using MTAG, displayed from left to right. Horizontal dashed grey lines indicate genome-wide significance threshold (*P* < 5.0 × 10^−8^). Dots colored in red indicates genome-wide significant loci. Asterisks indicate the novel findings

The results of single-disease association studies are shown in Fig. 1 and Table 2. The lambda values are compared in Supplementary Table 1. All variants are based on hg19. Previously reported variants are summarized in Supplementary Tables 2–6, and suggestive associations are summarized in Supplementary Tables 7–11. Functional annotations of the identified risk variants obtained by searching through HaploReg v4.1 are summarized in Supplementary Table 12. Annotations obtained by GARFIELD are shown in Supplementary Figs. 1–8. For UF, we identified four loci (rs7412010, chr1:g.22436446 G > C at 1p36, *CDC42/WNT4*, *P* = 1.2 × 10^−12^; rs12415148, chr10:g.105680586 T > C at 10q24, *STN1* (*OBFC1*), *P* = 3.5 × 10^−10^; rs12225799, chr11:g.241124 C > G at 11p15, *BET1L/*RIC8A, *P* = 1.1 × 10^−21^; rs17332320, chr22:g.40711620 G > T at 22q31, *TNRC6B*, *P* = 1.6 × 10^−12^; all *P*-values are of BOLT-LMM results). When the same variants were included in our results, the direction of effect of previously reported variants were concordant. For OC, we identified two loci (rs79219469, chr16:g.54587853 C > T at 16q12, *LINC02183*, *P* = 3.3 × 10^−8^; rs567534295, chr17:g.41200107 C > T at 17q21, *BRCA1*, *P* = 3.1 × 10^−8^; all *P*-values are of BOLT-LMM results). Of the previously reported variants, most have directionally concordant results. However, rs58722170, chr1:g.38096421 G > C at 1p34, *RSPO1*, rs2165109, chr2:g.111818658 A > C at 2q13, *ACOXL*, rs7953249, chr12:g.121403724 G > A at 12q24, *HNF1A-AS1*, and rs183211, chr17:g.44788310 G > A at 17q21, *NSF*, reported for its association with high-grade serous OC, and rs752590, chr2:g.113972945 A > G at 2q14, *PAX8-AS1*, rs112071820, chr3:g.138849113_138849114insGATTCAGAATCCA at 3q23, *MRPS22*, and rs688187, chr19:g.39732752 G > A at 19q13, *IFNL3*, reported for its association with mucinous OC, had directionally opposite effect in our samples [7]. Because we have not stratified the cases by the histopathological subtypes, difference in proportions of subtypes may explain this discordance. For UCC, we identified three loci (rs140668832, chr6:g.30479914 A > T at 6p22, the MHC region, *P* = 2.9 × 10^−10^; rs150806792, chr11:g.2179342 C > T at 11p15, *INS-IGF2*, *P* = 4.9 × 10^−8^; rs140991990, chr17:g.70097851 A > G at 17q24, *SOX9*, *P* = 3.3 × 10^−8^; all *P*-values are of BOLT-LMM results). For all the previously reported variants, the direction of effect was concordant. Out of these detected loci, two (rs79219469:C > T at 16q12, and rs567534295:C > T at 17q21) for OC [7], and two (rs150806792:C > T at 11p15, and rs140991990:A > G at 17q24) for UCC [9, 10] are novel associations in the context of GWAS [2–10]. The variant rs79219469:C > T may affect expression status of several genes by altering Gfi1 and Irf motifs, of which, Gfi1 is reported to have functions in oncogenesis. The variants rs150806792:C > T and rs140991990:A > G are reported as enhancer and promoter histone marks in several tissues which are not relevant to uterine cervix, however, alterations in Ets and GATA motifs, for example, may result in transcriptional activity of nearby genes. (Supplementary Table 12). For all associations, analysis under the linear mixed model using BOLT-LMM revealed more significant associations than the usual logistic regression model using mach2dat (e.g., rs7412010:G > C at 1p36 associated with UF showed *P*-values of 2.0 × 10^−11^ in mach2dat and 1.2 × 10^−12^ in BOLT-LMM). Generally, BOLT-LMM showed more significant associations than incorporating correlations among five GWAS estimates using MTAG, however, MTAG showed the most significant association at one locus, rs17332320:G > T at 22q31. We could not detect significant associations in endometriosis and UEC with any methods. Of the previously reported variants associated with endometriosis, most had the directionally concordant effect except for rs1250241, chr2:g.216 295312 T > A at 2q35, *FN1*, rs517875, chr3:g.174350886 C > A at 3q13, *RAP1BP2*, and rs13271465, chr8:g.17282411 T > C at 8p22, *MTMR7/ADAM24P*. Of the previously reported variants associated with uterine endometrial cancer, most had directionally concordant effect, however, rs1679014, chr9:g.22207037 T > C at 9p21, *CDKN2A/CDKN2B*, and rs2498796, chr14:g.105243220 G > A at 14q32, *AKT1*, had directionally discordant or inconsistent effect in our analysis.

**Table 2:** Genetic variants significantly associated with gynecologic diseases

Disease	SNP^a^	Chr	Position (bp)^b^	Genes	Ref*/*Alt^c^	AltAllele Freq^d^	Imputation Rsq	mach2dat		BOLT-LMM		MTAG		Novel loci
								OR (95% CI)	*P*	OR (95% CI)	*P*	OR (95% CI)	*P*	
Uterine fibroid	rs7412010	1	22 436 446	*CDC42, WNT4*	G*/*C	0.58	0.98	1.19 (1.13–1.24)	2.0 × 10^−11^	1.15 (1.10–1.19)	1.2 × 10^−12^	1.03 (1.02–1.05)	5.4 × 10^−8^	
	rs12415148	10	105 680 586	*STN1* (*OBFC1*)	T*/*C	0.078	0.97	1.24 (1.14–1.35)	8.6 × 10^−7^	1.25 (1.16–1.33)	3.5 × 10^−10^	1.05 (1.03–1.07)	2.0 × 10^−5^	
	rs12225799	11	241 124	*BET1L, RIC8A*	C*/*G	0.13	0.99	0.71 (0.65–0.76)	1.7 × 10^−17^	0.76 (0.72–0.80)	1.1 × 10^−21^	0.93 (0.91–0.94)	4.0 × 10^−17^	
	rs17332320	22	40 711 620	*TNRC6B*	G*/*T	0.36	1.00	1.18 (1.13–1.24)	4.4 × 10^−11^	1.15 (1.10–1.19)	1.6 × 10^−12^	1.05 (1.03–1.06)	3.9 × 10^−13^	
Ovarian cancer	rs79219469	16	54 587 853	*LINC02183*	C*/*T	0.039	0.94	1.85 (1.48–2.32)	6.9 × 10^−8^	2.25 (1.69–2.99)	3.3 × 10^−8^	1.10 (1.06–1.14)	1.5 × 10^−7^	*
	rs567534295	17	41 200 107	*BRCA1*	C*/*T	0.012	0.79	2.83 (1.94–4.12)	5.7 × 10^−8^	4.75 (2.73–8.24)	3.1 × 10^−8^	1.19 (1.12–1.26)	4.6 × 10^−8^	*
Uterine cervical cancer	rs140668832	6	30 479 914	the MHC region	A*/*T	0.11	0.95	1.73 (1.45–2.05)	5.8 × 10^−10^	1.92 (1.57–2.35)	2.9 × 10^−10^	1.07 (1.05–1.09)	1.8 × 10^−9^	
	rs117670375	6	30 687 472	the MHC region	C*/*T	0.11	0.94	1.73 (1.46–2.06)	5.2 × 10^−10^	1.92 (1.57–2.35)	3.0 × 10^−10^	1.07 (1.05–1.09)	1.9 × 10^−9^	
	rs150806792	11	2 179 342	*INS-IGF2*	C*/*T	0.012	0.92	2.79 (1.91–4.06)	1.1 × 10^−7^	4.96 (2.79–8.83)	4.9 × 10^−8^	1.19 (1.12–1.26)	8.3 × 10^−8^	*
	rs140991990	17	70 097 851	*SOX9*	A*/*G	0.015	0.72	2.76 (1.89–4.05)	1.8 × 10^−7^	4.94 (2.80–8.71)	3.3 × 10^−8^	1.16 (1.10–1.23)	6.8 × 10^−8^	*

The underlined odds ratios (OR) and *P*-values indicate the analytic method which showed the most significant association for each SNP

Among the three methods, mach2dat, BOLT-LMM, and MTAG. OR in BOLT-LMM is adjusted using case fraction u, using the formula; log(OR) = *β/*(u × (1-u))

^a^Variants significantly associated with gynecologic diseases

^b^Based on hg19

^c^Reference (Ref) and alternative (Alt) alleles on forward strand

^d^Alternative allele frequency among control subjects

There were five associations that reached genome-wide significance by BOLT-LMM but not by mach2dat; rs12415148:T > C at 10q24 for UF, rs79219469:C > T at 16q12 and rs567534295:C > T at 17q21.31 for OC, and rs150806792:C > T at 11p15 and rs140991990:A > G at 17q24 for UCC. Of these, four were low-frequency variants (1% < MAF < 5%). Although some associations were also detected as significant by MTAG, there were no variants that surpassed the genome-wide significance only by MTAG.

### Joint analysis of all the cases and controls, random-effect meta-analysis of single disease GWAS, and reverse regression analysis

We first performed two additional analyses; (i) joint analysis of all the combined cases of the five gynecologic diseases versus shared controls and (ii) random-effect meta-analysis of five single-disease association studies correcting for the overlapping samples using RE2C.

**Fig. 2:**
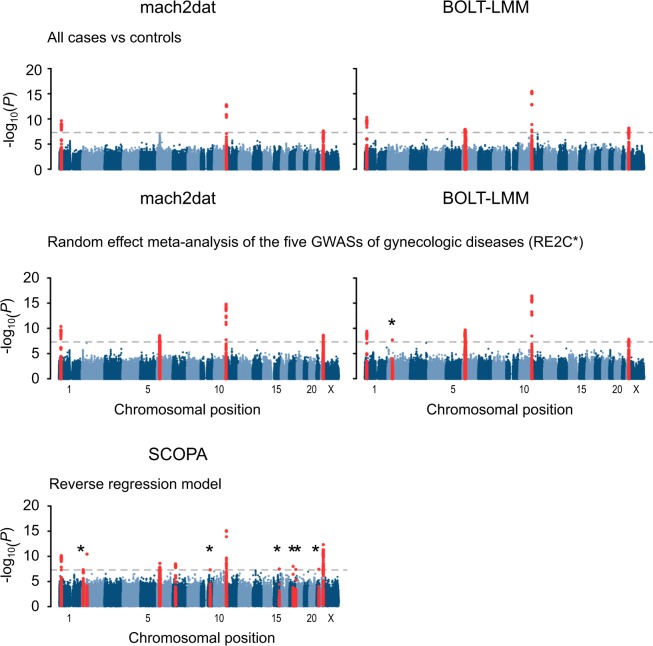
Manhattan plots of meta-analysis of the five GWASs of gynecologic diseases. Manhattan plots of association *P*-values of all cases versus shared controls (top), cross-trait random effect meta-analysis of the five GWASs of gynecologic diseases controlling for the overlapping samples using RE2C (middle), and the reverse regression analysis using SCOPA (bottom). Analyses under the logistic regression model using mach2dat and under the linear mixed model using BOLT-LMM are displayed from left to right. Horizontal dashed grey lines indicate genome-wide significance threshold (*P* < 5.0 × 10^−8^). Dots colored in red indicates genome-wide significant loci. An asterisk indicates the novel findings

Joint analysis of all cases versus shared controls yielded four associations that surpassed the significance threshold (rs7412010:G > C at 1p36, rs12225799:C > G at 11p15, rs17332320:G > T at 22q13, and the MHC region at 6p22, Fig. 2 and Table 3). Out of the four associations, three (rs7412010:G > C at 1p36, rs12225799:C > G at 11p15, and rs17332320:G > T at 22q13) were identical to those detected in single disease analysis of UF, and were detected both by mach2dat and BOLT-LMM, while the remaining one, the MHC region, was identical to that found in single disease analysis of UCC, and was significant only in the analysis by BOLT-LMM. For all associations that surpassed the genome-wide significance, BOLT-LMM showed more significance than mach2dat, as was shown in the single-disease associations.

**Table 3:** Meta-analysis results of the five GWASs of gynecologic diseases

SNP^a^	Chr	Position (bp)^b^	Genes	Ref*/*Alt^c^	Alt Allele Freq^d^	Imputation Rsq	mach2dat				BOLT-LMM				Novel loci
							All cases vs controls		RE2C		All cases vs controls		RE2C		
							OR (95% CI)	*P*	*P*	*I*^2^ (%)^e^	OR (95% CI)	*P*	*P*	*I*^*2*^ (%)^e^	
rs7412010	1	22 436 446	*CDC42, WNT4*	G*/*C	0.58	0.98	1.14 (1.10–1.19)	2.3 × 10^−10^	4.3 × 10^−11^	77.8	1.12 (1.08–1.15)	5.0 × 10^−11^	3.7 × 10^−10^	91.7	
rs937380553	2	52 063 361	*LOC730100*	A*/*G	0.014	0.94	1.22 (1.03–1.45)	2.1 × 10^−2^	8.9 × 10^−8^	83.0	1.16 (1.01–1.33)	3.6 × 10^−2^	2.0 × 10^−8^	77.4	*
rs202217993	6	29 386 975	the MHC region	GT*/*G	0.10	0.95	1.20 (1.12–1.28)	2.7 × 10^−7^	1.8 × 10^−8^	69.2	1.18 (1.11–1.25)	1.2 × 10^−8^	1.8 × 10^−9^	76.2	
rs17179851	6	29 924 440	the MHC region	T*/*C	0.25	0.93	1.14 (1.08–1.19)	8.8 × 10^−8^	9.3 × 10^−8^	44.3	1.11 (1.07–1.15)	6.8 × 10^−8^	6.8 × 10^−7^	74.1	
rs117670375	6	30 687 472	the MHC region	C*/*T	0.11	0.94	1.18 (1.10–1.26)	1.0 × 10^−6^	2.8 × 10^−9^	81.3	1.16 (1.10–1.22)	1.3 × 10^−7^	2.0 × 10^−10^	79.2	
rs12225799	11	241 124	*BET1L, RIC8A*	C*/*G	0.13	0.99	0.78 (0.73–0.84)	1.5 × 10^−13^	2.1 × 10^−15^	83.7	0.81 (0.78–0.86)	3.3 × 10^−16^	3.7 × 10^−17^	95.3	
rs141244868	11	244 817	*BET1L, RIC8A*	GA*/*G	0.13	0.98	0.78 (0.73–0.84)	1.9 × 10^−13^	1.6 × 10^−15^	84.0	0.81 (0.78–0.86)	4.7 × 10^−16^	4.2 × 10^−17^	95.3	
rs112251865	22	40 665 225	*TNRC6B*	C*/*T	0.36	0.99	1.12 (1.08–1.17)	3.0 × 10^−8^	2.3 × 10^−9^	75.5	1.10 (1.07–1.14)	7.6 × 10^−9^	3.3 × 10^−8^	91.4	
rs17332320	22	40 711 620	*TNRC6B*	G*/*T	0.36	1.00	1.12 (1.08–1.17)	2.4 × 10^−8^	2.9 × 10^−7^	77.3	1.10 (1.07–1.14)	6.7 × 10^−9^	1.5 × 10^−8^	91.6	

The underlined odds ratios (OR) and *P*-values indicate the analytic method which showed the most significant association for each SNP among the four methods, joint analysis of all cases versus shared controls and random-effect meta-analysis using mach2dat and BOLT-LMM, respectively

OR in BOLT-LMM is adjusted using case fraction u, using the formula; log(OR) = β*/*(u × (1-u)) RE2C does not produce OR

^a^Variants significantly associated with gynecologic diseases.

^b^Based on hg19

^c^Reference (Ref) and alternative (Alt) alleles on forward strand

^d^Alternative allele frequency among control subjects

^e^*I*^*2*^ statistics were calculated using Metasoft (v2.0.1)

Random-effect meta-analysis of the five GWASs of gynecologic diseases using RE2C detected one additional novel association in chromosome 2 (rs937380553, chr2:g.52063361 A > G at 2p16, *LOC730100*, *P* = 2.0 × 10^−8^ in RE2C* with BOLT-LMM results, Fig. 2 and Table 3). This detection was achieved only with the use of summary statistics derived from BOLT-LMM. This locus was nominally associated with endometriosis, OC, and UEC (*P* = 2.9 × 10^−5^, 5.5 × 10^−4^, and 1.3 × 10^−4^, in endometriosis, OC, and UEC, respectively, Supplementary Table 13).

We next performed reverse regression analysis using SCOPA. When the MHC region is counted as one locus, we identified 12 loci in association with gynecologic diseases (Fig. 2 and Table 4). Of these, seven loci were best explained when the multiple gynecologic diseases were combined in the regression model. In addition to the three novel loci identified in single disease GWAS and meta-analysis, three loci (rs73494486, chr9:g.101341851 C > T at 9q22, *GABBR2*, *P* = 4.8 × 10^−8^; rs145152209, chr15:g.84077212 A > G at 15q25, *SH3GL3/BNC1*, *P* = 3.3 × 10^−8^; and rs147427629, chr21:g.40419321 G > A at 21q22, *LOC107985484*, *P* = 3.8 × 10^−8^) were novel findings. The top SNPs in the previously identified loci were almost the same as those detected in single disease GWAS and meta-analysis (Tables 2–4). Annotations obtained by searching through HaploReg v4.1 are summarized in Supplementary Table 12.

**Table 4:** Summary statistics of the detected variants in SCOPA

SNP^a^	Chr	Position (bp)^b^	Genes	Ref / Alt^c^	AltAlleleFreq^d^	Imputation Rsq	Diseases in the best model	*P*	Novel loci
rs7412010	1	22 436 446	*WNT4*, *CDC42*	C*/*G	0.42	0.98	UF	7.0E–11	
rs6432216	2	11 702 960	*GREB1*	T*/*C	0.27	1.00	UF + Endometriosis	4.6E–08	
rs937380553	2	52 063 361	*LOC730100*	A*/*G	0.014	0.94	Endometriosis + OC + UEC	3.6E–11	*
rs9257985	6	29 652 253	The MHC region	A*/*G	0.14	1.00	UF + UCC	1.5E–08	
rs117670375	6	30 687 472	The MHC region	C*/*T	0.11	0.95	UCC	1.4E–08	
rs2507968	6	31 372 718	The MHC region	G*/*A	0.74	0.95	UF + UCC	2.5E–09	
rs9271215	6	32 579 277	The MHC region	C*/*T	0.53	0.90	UF + UCC	2.4E–08	
rs116832992	7	31 784 755	*PDE1C*	T*/*C	0.059	0.91	Endometriosis + UCC	3.2E–09	
rs73494486	9	101 341 851	*GABBR2*	C*/*T	0.14	0.95	UF + OC	4.8E–08	*
rs138060871	11	241 284	*BET1L*, *RIC8A*	G*/*A	0.085	0.86	UF	7.8E–16	
rs145152209	15	84 077 212	*SH3GL3*, *BNC1*	A*/*G	0.075	0.96	UF	3.3E–08	*
17:41200107	17	41 200 107	*BRCA1*	C*/*T	0.012	0.79	OC	1.0E–08	*
17:70097851	17	70 097 851	*SOX9*	A*/*G	0.016	0.73	UCC	3.9E–08	*
rs147427629	21	40 419 321	*LOC107985484*	G*/*A	0.023	0.84	OC + UEC	3.8E–08	*
rs17332320	22	40 711 620	*TNRC6B*	G*/*T	0.36	1.00	UF + Endometriosis	4.7E–13	

*UF* Uterine fibroid, *OC* ovarian cancer, *UEC* uterine endometrial cancer, *UCC* uterine cervical cancer

^a^Variants significantly associated with gynecologic diseases

^b^Based on hg19

^c^Reference (Ref) and alternative (Alt) alleles on forward strand

^d^Alternative allele frequency among control subjects

^e^SCOPA produces *P*-value of the best model

When comparing the five patterns of the analyses, namely, combination of two conjoining methods (joint analysis of all cases versus shared controls, and random-effect meta-analysis of the five GWAS correcting for the overlapping subjects), and two analytic methods (the usual logistic model using mach2dat, and the linear mixed model using BOLT-LMM), and reverse regression model, the association results were heterogeneous. These results would suggest that applying multiple methods would offer more opportunity to detect significantly associated loci.

### MHC region

Within those detected loci, the variants showing the most significant associations are almost identical among the analytic methods. However, the MHC region at chromosome 6, which surpassed genome-wide significance in single-disease GWAS of UCC, joint analysis, and random-effect meta-analysis, showed the most significant associations at different variants among the methods as previously suggested [30]. Fine-mapping and identification of causal variants of the MHC region by high resolution HLA imputation would be warranted [30, 35].

### BRCA1

*BRCA1* is a tumor suppressor gene well-known from the pedigree studies of familial breast and ovarian cancers. In this study, we identified the significant association with OC at this gene locus in the GWAS approach for the first time. While previous genetic studies mostly focused on coding variants of *BRCA1* [36], the associated variant, rs567534295:C > T, was the low-frequency noncoding variant (1% < MAF among controls < 5%), located within the intron between exon 22 and exon 23, and all the variants in moderate LD (*r*^2^ > 0.5) with rs567534295:C > T were not located within the coding region. Rs567534295:C > T is monomorphic in non-east Asian populations (in 1KGP phase3v5a), suggesting population-specific risk of the variant on OC. To make functional annotation of this low-frequency risk variant, we assessed the existing expression quantitative trait locus (eQTL) databases (GTEx, HGVD, Ishigaki et al. [37]) and applied artificial intelligence(AI)-based prediction algorithm on variant functions (ExPecto [38]). However, known eQTL databases and annotation tools do not contain these variants, and AI-based prediction showed that *BRCA1* expression was not affected by the variant rs567534295:C > T, suggesting the contribution of undetermined mechanisms to the pathophysiology of OC.

### Heritability and genetic correlation

The disease prevalence used for estimating heritability is shown in Table 1. GCTA-GREML applied to the genotype matrix of each disease produced very stringent results; heritability estimated from genotyped SNPs (*h*^*2*^_*SNP*_ in Table 1) ranged from 0.0260 for ovarian cancer to 0.220 for endometriosis (Table 1). We observed that the *h*^*2*^_*SNP*_ is about the fifteenth to the two thirds of the disease heritability reported in epidemiological studies (*h*^*2*^_*g*_ in Table 1).

**Fig. 3:**
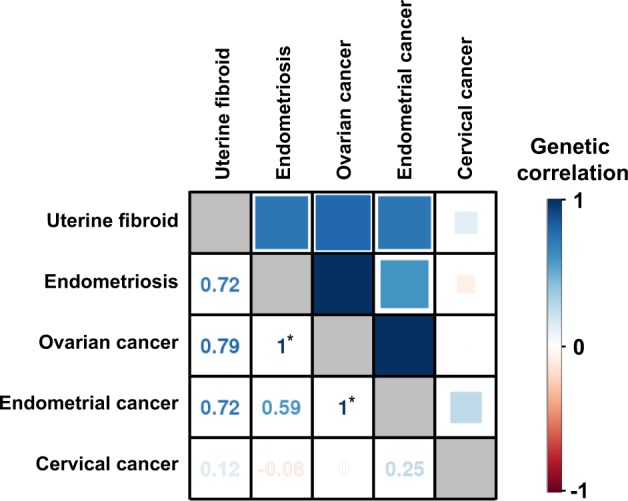
Cross-trait evaluation of genetic correlation among five gynecologic diseases. Genetic correlations among five gynecologic diseases calculated under the linear mixed model by Haseman–Elston regression using PCGC-s. Correlation is expressed by the color and size of square on the right upper triangle, while represented in digits on the left lower triangle. Asterisks indicate that the real output value exceeded one but was set to one for display purpose

Genetic correlations among the five gynecologic diseases under the linear mixed model, calculated directly from the genotyped SNPs, are shown in Fig. 3. All correlations among the four gynecologic diseases except for UCC were directionally positive, and stronger correlations were detected between endometriosis and OC (*r*_g _≥ 1.00), OC and UEC (*r*_g _≥ 1.00), and UF and OC (*r*_g _= 0.79). While relatively weaker and sometimes negative correlations were depicted between UCC and the four other gynecologic diseases (*r*_g _= −0.08–0.25, Fig. 3).

## Discussion

In this study, we detected nine significant associations with three of the five gynecologic diseases. Four out of the nine associated loci were identified by applying the linear mixed model but not by the usual logistic regression model. For example, rs12415148:T > C at *STN1/SLK* associated with UF is a known risk locus for UF but was not detected by the usual logistic regression model approach with the current sample size. This supports the application of the linear mixed model approach to the case-control studies to enhance the detection of genetic associations.

We identified two novel loci associated with OC, rs79219469:C > T and rs567534295:C > T. We compared our results with the publicly available summary statistics from the largest European OC GWAS. We found that chr16 variant rs79219469:C > T exists in the summary but its effect allele (*T*) frequency is only 0.0046, with its association *p*-value 0.84. We also looked for the chr17 variant rs567534295:C > T, however, this does not exist in the available summary (monomorphic in 1KG Europeans). We considered that disease risk of these variants were rather population-specific in Japanese (and east Asians).

We identified two novel loci associated with UCC, rs150806792:C > T and rs140991990:A > G. Rs150806792:C > T at *INS-IGF2* locus is suggested to be associated in the pathogenesis of UCC through the activation of insulin-like growth factor pathway, as reported in the cases of colorectal tumors [39]. Rs140991990:A > G is located at *SOX9* locus, which is a member of SOX family and its family member *SOX14* is reported to be involved in p53 signaling pathway in a UCC cell line [40]. Also, inhibition of *SOX9* is reported to increase radiosensitivity in gastrointestinal cancer [41]. These suggest the roll of *SOX9* in the pathogenesis of UCC.

Of the nine identified associations, three top variants; rs567534295:C > T for OC, and rs150806792:C > T and rs140991990:A > G for UCC, are variants found only in Japanese and/or East Asians with rare to low-frequency but not observed in other populations, according to 1KGP phase 3v5a data. These three were also the variants better detected under the mixed model. Of note, the well-known *BRCA1* locus, encompassing the low frequency noncoding variant of rs567534295:C > T, was shown to be significantly associated with OC for the first time in the context of GWAS [7]. We note that risk identification of rs567534295:C > T could have been achieved only by using the large population-specific imputation reference panel [25, 26], with adequate GWAS sample size, and by using ingenious analytical methods. While the GARFIELD analysis suggested functional annotations of genetics of the phenotypes, cell specificity observed in the results were still relatively nonspecific. Further approaches would be warranted to further elucidate undetermined disease mechanisms.

In the joint analysis, since sample sizes are different among the diseases, signals are most likely to be driven by the disease with the largest samples. As expected, three signals were concordant with those detected in UF GWAS, having the largest samples among the five GWASs, however, signals in the MHC region were also significant under the linear mixed model, which were concordant with those detected in UCC GWAS, having the smallest samples. This showed that strength of the signals in joint analysis can be either amplified or attenuated depending on the sample sizes and correlations among the diseases at the signal of the interest.

A novel association was also discovered by random-effect meta-analysis of the five GWASs adjusting for the overlapping samples. The top associated SNP rs937380553:A > G at 2p16 locus is located within noncoding *LOC730100* gene. The function of this lncRNA is not well investigated and further studies are required to elucidate its contribution to the pathogenesis of gynecologic diseases, especially, possibly shared effect among endometriosis, OC, and UEC. We also applied a novel approach, MTAG, which incorporates correlations among multiple GWAS estimates to enhance detection, to gynecologic diseases. In some, but not all, MTAG results were the most significant among those of the usual logistic regression model and the linear mixed model. Therefore, by applying multiple analytic methods, we can have more opportunity to identify novel associations.

In the reverse regression analysis, we identified three additional novel associations. Rs73494486:C > T at *GABBR2* locus is associated with combined phenotypes of UF and OC. This locus has neither been reported in the GWAS of UF or OC, however, *GABBR2* is suggested to have an important role in EGFR signaling through the ERK1/2 pathway, as reported in lung adenocarcinoma [42]. Rs145152209:A > G at *SH3GL3/BNC1* locus is associated with UF. *SH3GL3* and *BNC1* are both neither reported in the context of UF, however, *SH3GL3* is reported as a colorectal cancer-associated gene [43], and *BNC1* is reported to have association with pancreatic cancer [44, 45]. These suggest that this locus may play an role in proliferative property of UF in some undetermined mechanisms. Rs147427629:G > A at *LOC107985484* locus is associated with OC + UEC. This noncoding RNA is not studied well, however, the similarity of OC and UEC supports the mutual pathophysiology conferred by this non-coding RNA.

We showed the genetic correlations under the mixed model among the five gynecologic diseases. As expected from the past genetic and epidemiologic studies [21–23], these correlations were mostly directionally positive. The correlation was stronger between OC and UEC, and between endometriosis and OC, compared with those between UCC and the other four gynecologic diseases, which was concordant with the epidemiological findings [1, 20–22]. Thinking of the pathophysiology, where UCC is mainly caused by infection of human papilloma viruses, which is distinct from other gynecologic diseases, and where some histological subtypes of OC very often co-occur with endometriosis [1], this result would be considered rational. Although genome-wide additive effects are correlated given the strong genetic correlation and similarity of SNP effect sizes among these gynecologic diseases, we could detect only one additional shared locus in the cross-trait meta-analysis. This was because many diseases have polygenic nature and most of the variants have too small effect sizes to be detected. By increasing sample sizes, more shared loci with small effects are expected to be detected. This encourages cross-trait meta-analysis to support improved power to detect shared loci as described above.

Strength of our study includes the followings: First, we conducted the GWASs with the largest sample sizes among Japanese population, which facilitated the detection of novel associations. Second, we performed association analyses under the linear mixed model and also applied MTAG. We demonstrated that application of the mixed model and/or integrating correlations among multiple diseases increased the power of detection. Third, we used the population-specific imputation reference panel, which contributed to the increased number of variants with higher accuracy, especially those specific to the Japanese population such as the risk noncoding variant at *BRCA1*. Fourth, we demonstrated that combining the GWASs of multiple diseases in a random-effect meta-analysis revealed a novel candidate association. Fifth, we revealed genetic correlations among multiple gynecologic diseases. Shared genetic etiology encourages the investigation of common pathophysiology of the related diseases.

Although our study is the largest GWAS in the Japanese population, our limitation includes the lack of replication study. This is the task for our future study.

In conclusion, we successfully identified nine significant genetic associations with three gynecologic diseases including four novel ones, by applying association analysis under the mixed model and incorporating correlations among multiple GWAS estimates. Further, cross-trait meta-analysis identified five loci including one novel association which is suggested to be a shared risk locus. We also disclosed genetic correlations among multiple gynecologic diseases. We propose to apply new methodologies to increase detection power, and cross-trait analysis to assess shared risks.

### URLs

OMIM, http://www.omim.org/

EIGENSOFT v6.1.4, https://www.hsph.harvard.edu/alkes-price/software/

PLINK 1.9b3.3, https://www.cog-genomics.org/plink2

Minimac3, https://genome.sph.umich.edu/wiki/Minimac

1000 Genome Project, http://www.internationalgenome.org/

Eagle, https://data.broadinstitute.org/alkesgroup/Eagle/

mach2dat, https://genome.sph.umich.edu/wiki/Mach2dat:_Association_with_MACH_output

Metasoft, http://genetics.cs.ucla.edu/meta/

GWAS catalog, https://www.ebi.ac.uk/gwas/

HaploReg v4.1, https://pubs.broadinstitute.org/mammals/haploreg/haploreg.php

GENCODE v19 (GRCh37.p13), https://www.gencodegenes.org/releases/19.html

GTEx, https://gtexportal.org/home/

HGVD, http://www.hgvd.genome.med.kyoto-u.ac.jp/

ExPecto, https://github.com/FunctionLab/ExPecto

## Supplementary information

Supplementary information

Supplementary Figures

Supplementary Tables
