# Supplementary material for: GWAS of five gynecologic diseases and cross-trait analysis in Japanese
Source: Eur J Hum Genet. 2019 Sep 5;28(1):95–107. doi: 10.1038/s41431-019-0495-1 (PMC6906293; doi:10.1038/s41431-019-0495-1)
Supplement: Supplementary file 1 — Supplementary information [file 41431_2019_495_MOESM1_ESM.docx]

Supplementary information

Supplemental figure 1

GARFIELD results: Chromatin states of five gynecologic diseases.

Supplemental figure 2

GARFIELD results: FAIRE of five gynecologic diseases.

Supplemental figure 3

GARFIELD results: Footprints of five gynecologic diseases.

Supplemental figure 4

GARFIELD results: Genic enrichment patterns of five gynecologic diseases.

Supplemental figure 5

GARFIELD results: Histone modifications of five gynecologic diseases.

Supplemental figure 6

GARFIELD results: Hotspots of five gynecologic diseases.

Supplemental figure 7

GARFIELD results: Peaks of five gynecologic diseases.

Supplemental figure 8

GARFIELD results: Tissue factor binding site enrichment of five gynecologic diseases.

Supplementary table 1

Lambda values of each GWAS.

Genomic inflation lambda values are listed. Lambda values are not available for RE2C* and SCOPA.

Supplementary Table 2

Summary statistics of the reported variants. Uterine fibroid.

Supplementary Table 3

Summary statistics of the reported variants. Endometriosis.

Supplementary Table 4

Summary statistics of the reported variants. Ovarian cancer.

Supplementary Table 5

Summary statistics of the reported variants. Uterine endometrial cancer.

Supplementary Table 6

Summary statistics of the reported variants. Uterine cervical cancer.

Supplementary Table 7

Suggestive associations in GWAS for uterine fibroid.

Supplementary Table 8

Suggestive associations in GWAS for endometriosis.

Supplementary Table 9

Suggestive associations in GWAS for ovarian cancer.

Supplementary Table 10

Suggestive associations in GWAS for uterine endometrial cancer.

Supplementary Table 11

Suggestive associations in GWAS for uterine cervical cancer.

Supplementary Table 12

Functional annotation of the detected variants.

HaploReg v4.1 results for the identified variants are listed.

Supplementary Table 13.

Summary statistics of the detected variants in each single disease GWAS.

Summary statistics of single disease GWAS for variants identified in joint analysis and meta-analysis.
