# Supplementary material for: GWAS of five gynecologic diseases and cross-trait analysis in Japanese
Source: Eur J Hum Genet. 2019 Sep 5;28(1):95–107. doi: 10.1038/s41431-019-0495-1 (PMC6906293; doi:10.1038/s41431-019-0495-1)
Supplement: Supplementary file 2 — Supplementary Figures [file 41431_2019_495_MOESM2_ESM.pptx]

## Slide 1
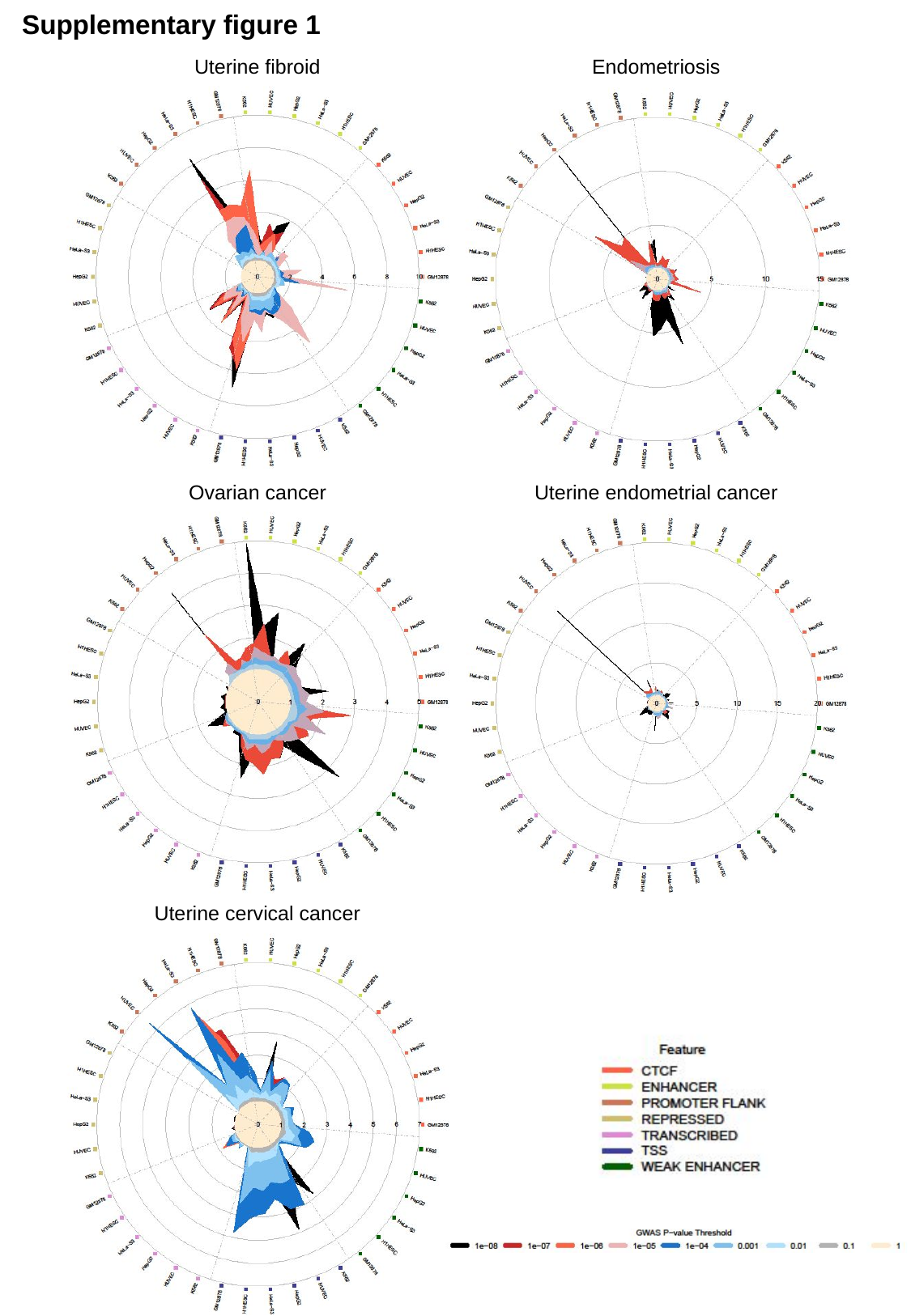

Supplementary figure 1
Uterine fibroid
Endometriosis
Ovarian cancer
Uterine endometrial cancer
Uterine cervical cancer

## Slide 2
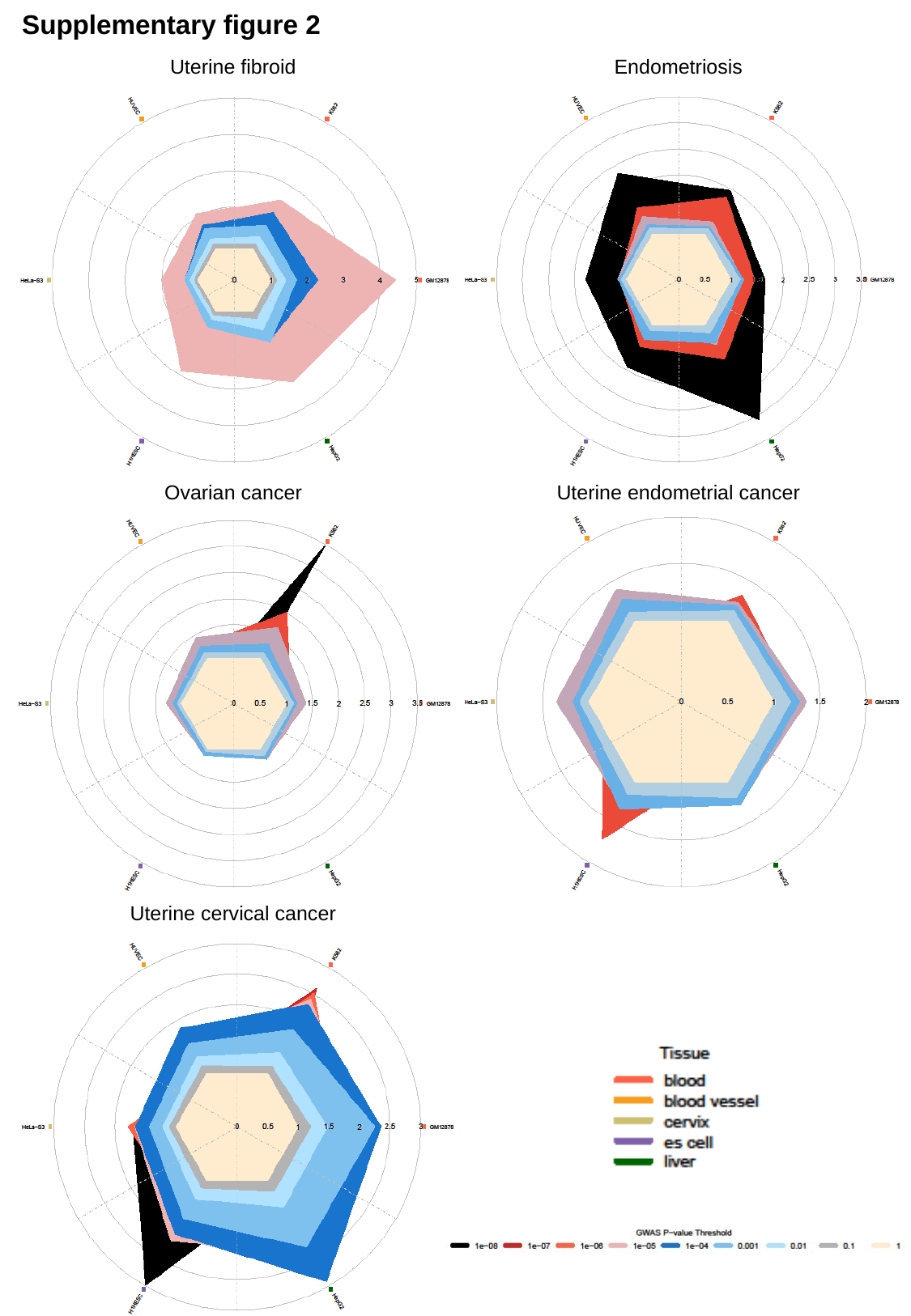

Supplementary figure 2
Uterine fibroid
Endometriosis
Ovarian cancer
Uterine endometrial cancer
Uterine cervical cancer

## Slide 3
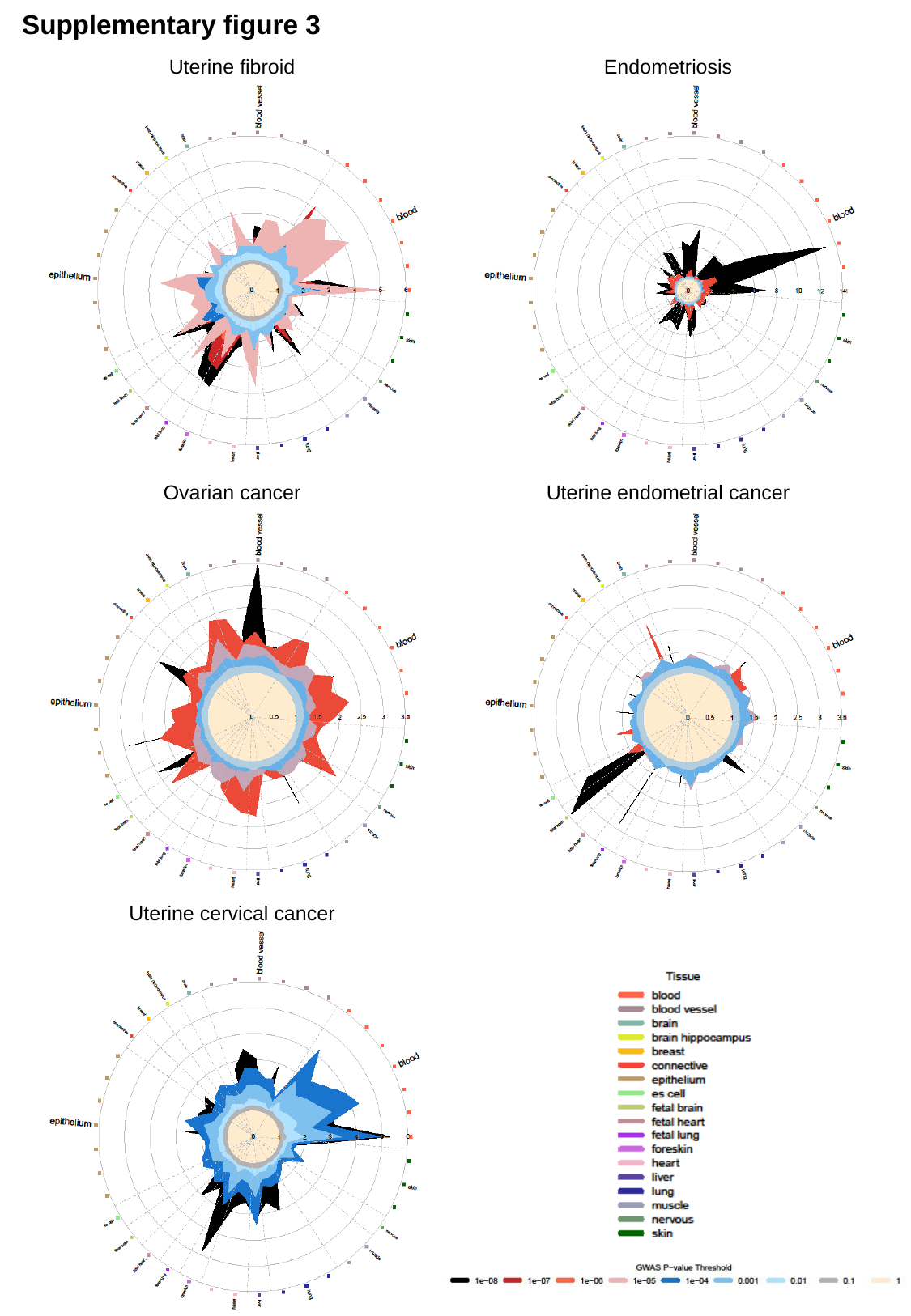

Supplementary figure 3
Uterine fibroid
Endometriosis
Ovarian cancer
Uterine endometrial cancer
Uterine cervical cancer

## Slide 4
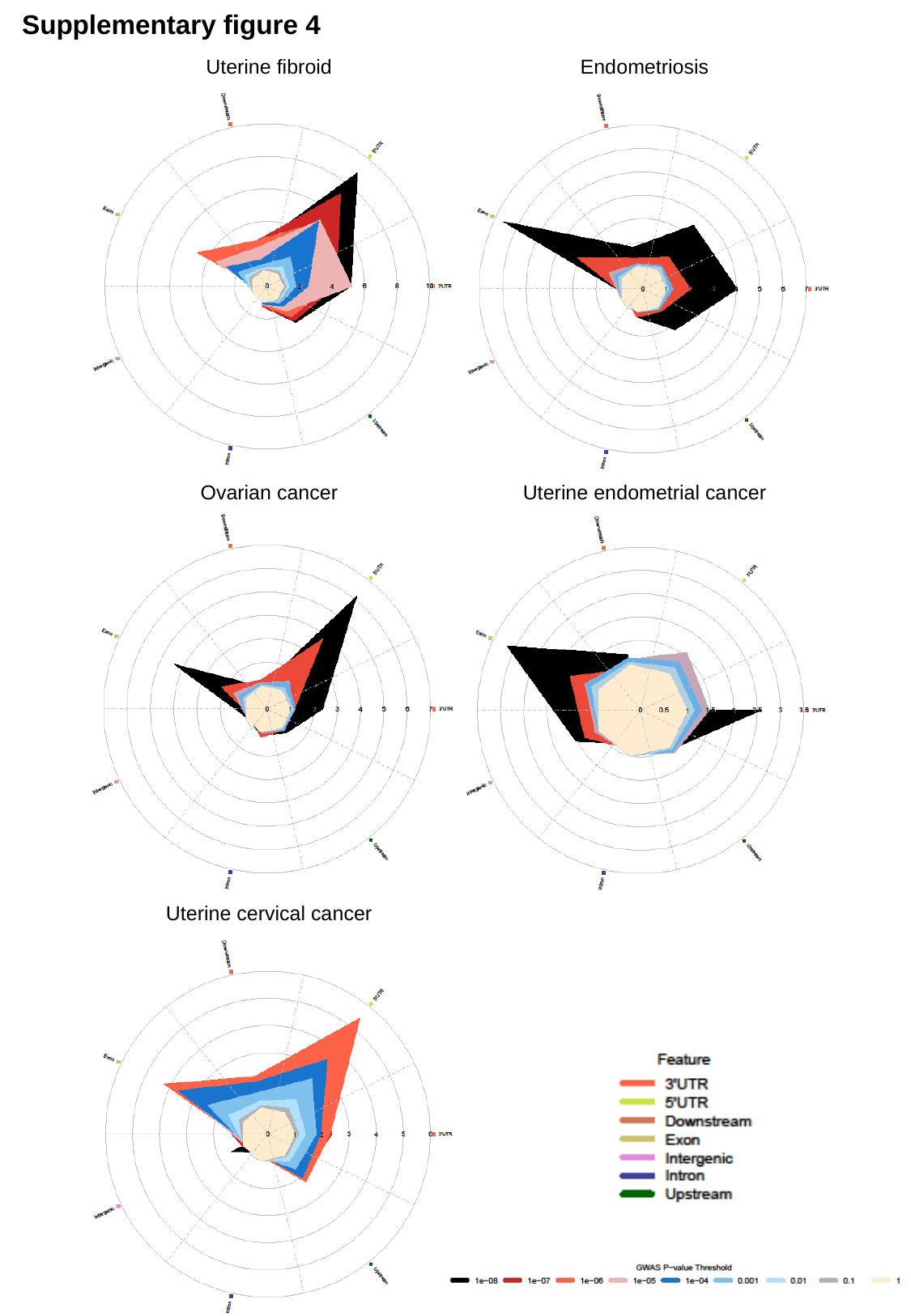

Supplementary figure 4
Uterine fibroid
Endometriosis
Ovarian cancer
Uterine endometrial cancer
Uterine cervical cancer

## Slide 5
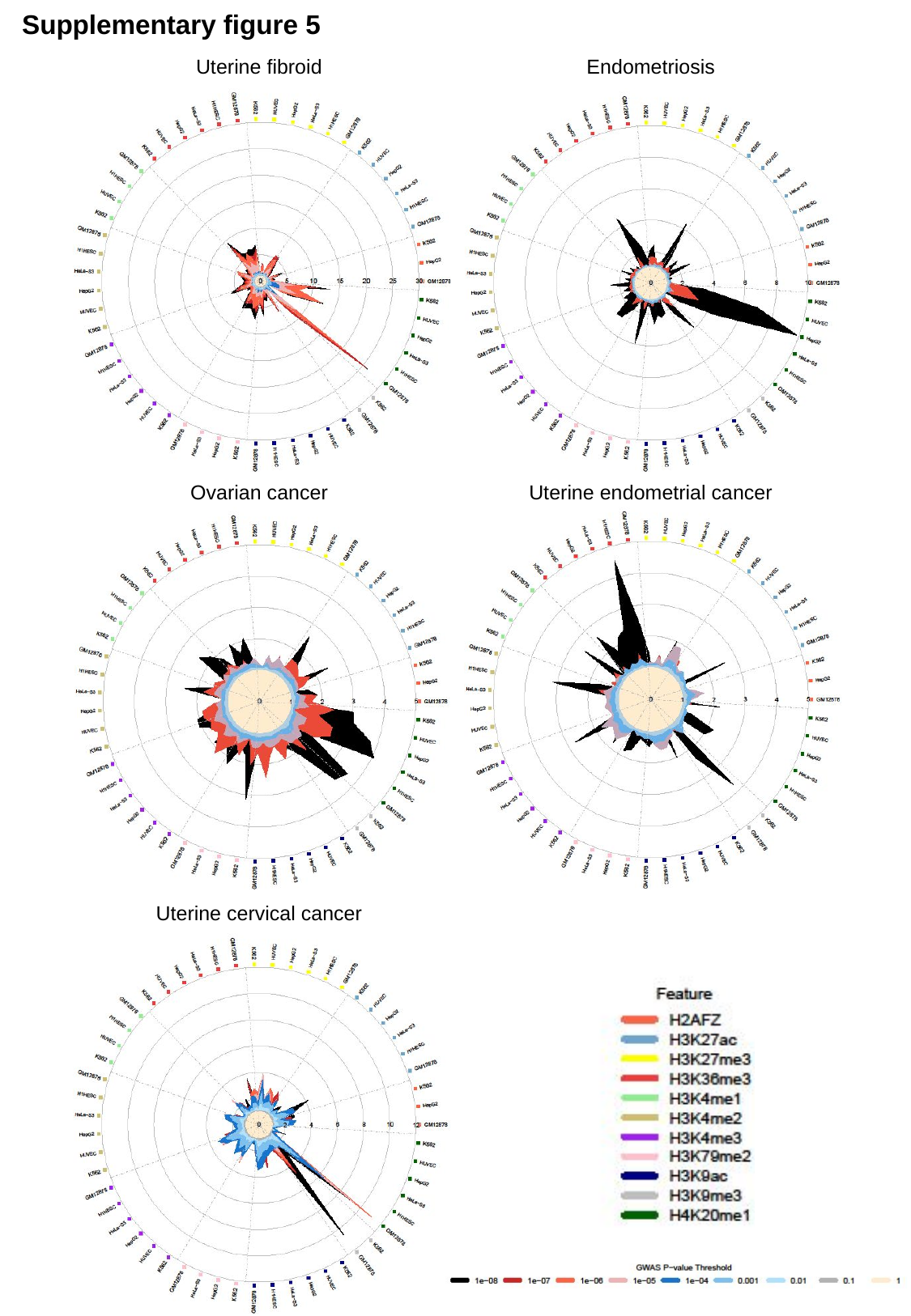

Supplementary figure 5
Uterine fibroid
Endometriosis
Ovarian cancer
Uterine endometrial cancer
Uterine cervical cancer

## Slide 6
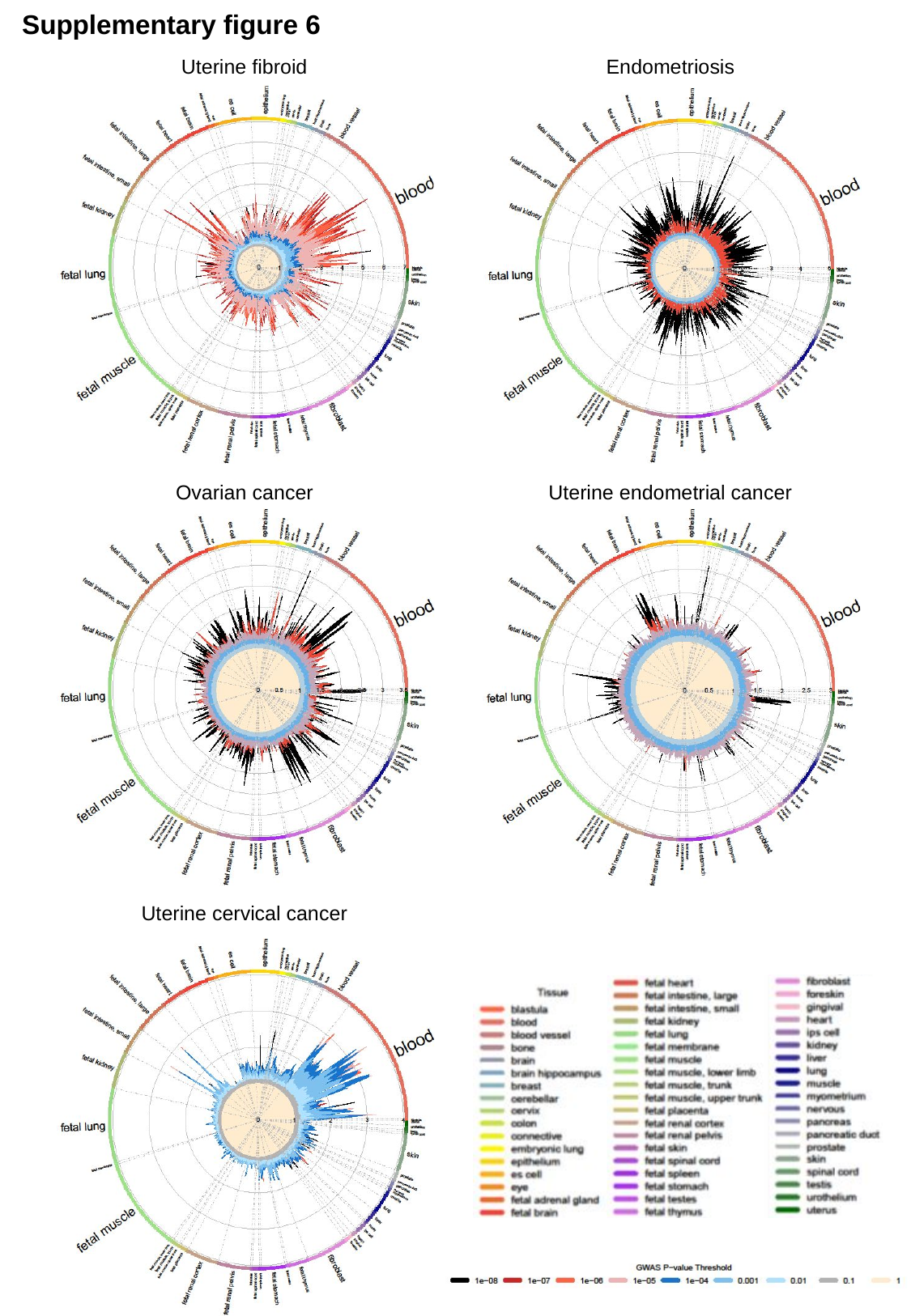

Supplementary figure 6
Uterine fibroid
Endometriosis
Ovarian cancer
Uterine endometrial cancer
Uterine cervical cancer

## Slide 7
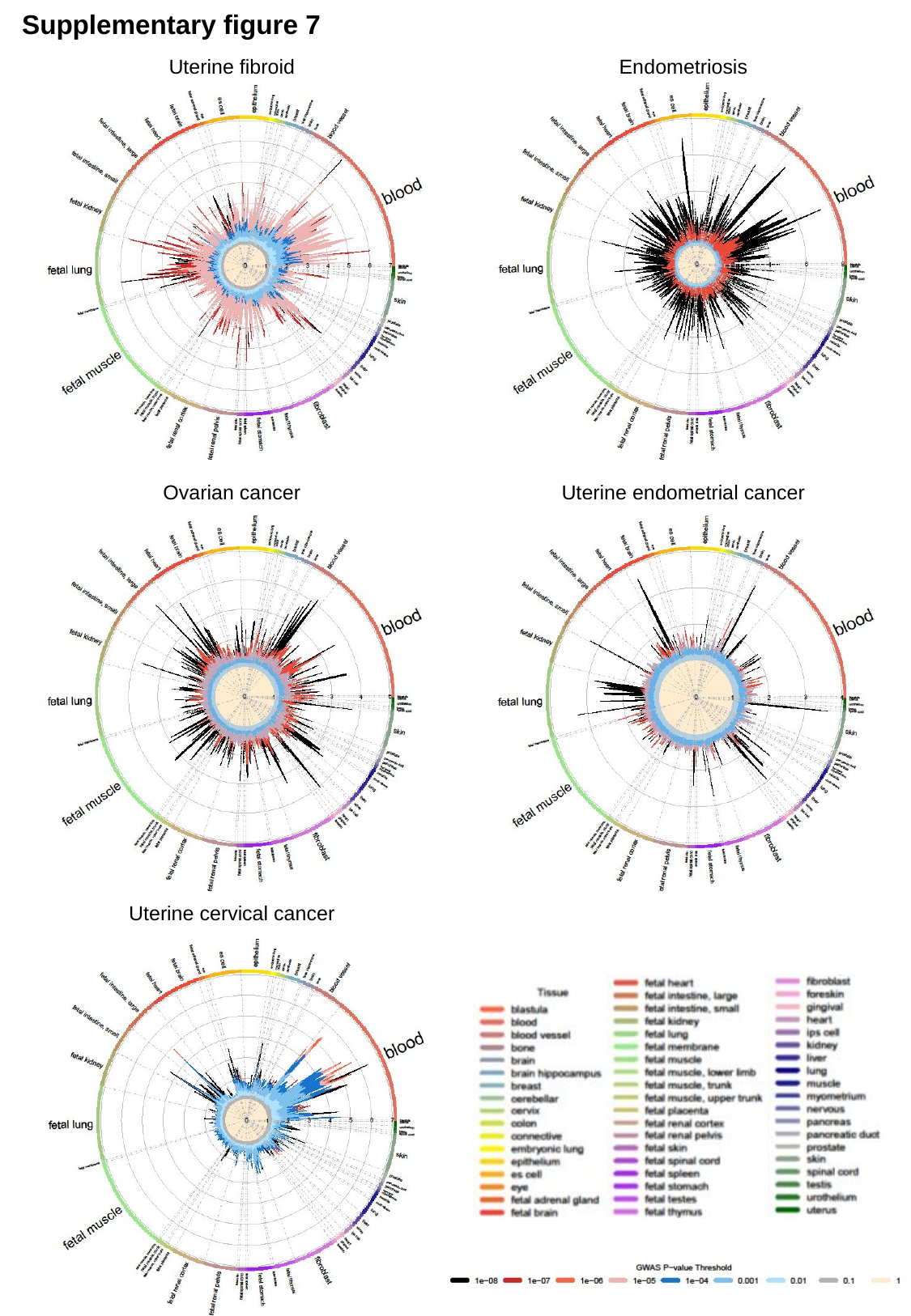

Supplementary figure 7
Uterine fibroid
Endometriosis
Ovarian cancer
Uterine endometrial cancer
Uterine cervical cancer

## Slide 8
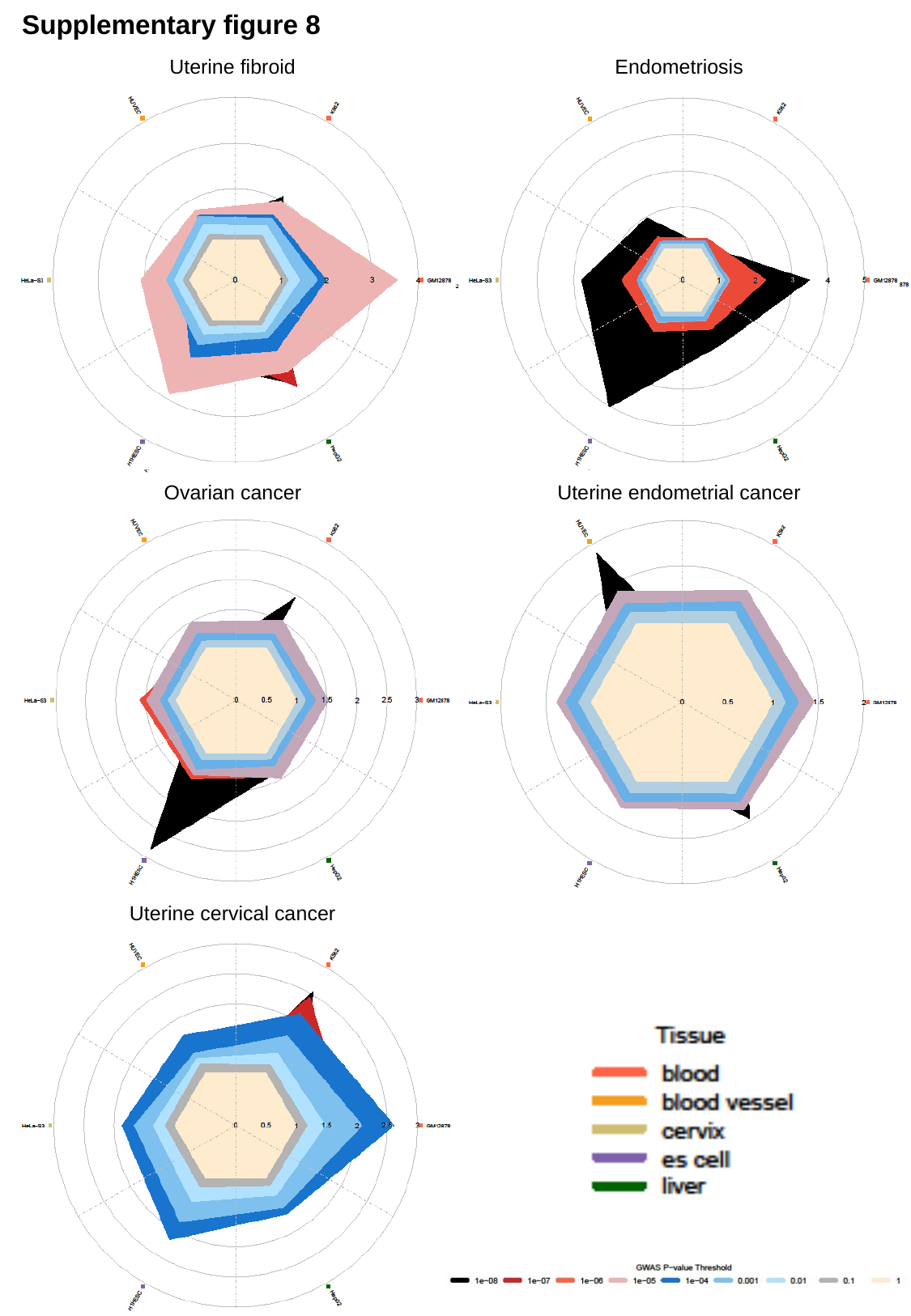

Supplementary figure 8
Uterine fibroid
Endometriosis
Ovarian cancer
Uterine endometrial cancer
Uterine cervical cancer
